# External validation of the Revised Cardiac Risk Index and update of its renal variable to predict 30-day risk of major cardiac complications after non-cardiac surgery: rationale and plan for analyses of the VISION study

**DOI:** 10.1136/bmjopen-2016-013510

**Published:** 2017-01-09

**Authors:** Pavel S Roshanov, Michael Walsh, P J Devereaux, S Danielle MacNeil, Ngan N Lam, Ainslie M Hildebrand, Rey R Acedillo, Marko Mrkobrada, Clara K Chow, Vincent W Lee, Lehana Thabane, Amit X Garg

**Affiliations:** 1 Lilibeth Caberto Kidney Clinical Research Unit, London Health Sciences Centre, London, Ontario, Canada; 2 Department of Clinical Epidemiology & Biostatistics, McMaster University, Hamilton, Ontario, Canada; 3 Department of Medicine, McMaster University, Hamilton, Ontario, Canada; 4 Population Health Research Institute, Hamilton, Ontario, Canada; 5 Department of Otolaryngology Head & Neck Surgery and Department of Oncology, Western University, London, Ontario, Canada; 6 Institute for Clinical Evaluative Sciences, London, Ontario, Canada; 7 Division of Nephrology, University of Alberta, Edmonton, Alberta, Canada; 8 Department of Medicine, Western University, London, Ontario, Canada; 9 The George Institute for Global Health, Sydney Medical School, University of Sydney, Sydney, New South Wales, Australia; 10 Department of Cardiology, Westmead Hospital, Sydney, New South Wales, Australia; 11 Department of Renal Medicine, Westmead Hospital, Western Sydney Local Health District, Sydney, New South Wales, Australia; 12 Centre for Transplant and Renal Research, Westmead Institute for Medical Research, University of Sydney, Westmead, New South Wales, Australia; 13 Centre for Evaluation of Medicines and Biostatistics Unit, St Joseph's Healthcare, Hamilton, Ontario, Canada; 14 Departments of Paediatrics and Anaesthesia, McMaster University, Hamilton, Ontario, Canada; 15 Department of Epidemiology and Biostatistics, Western University, London, Ontario, Canada

**Keywords:** Risk prediction, perioperative medicine, cardiac events, SURGERY

## Abstract

**Introduction:**

The Revised Cardiac Risk Index (RCRI) is a popular classification system to estimate patients' risk of postoperative cardiac complications based on preoperative risk factors. Renal impairment, defined as serum creatinine >2.0 mg/dL (177 µmol/L), is a component of the RCRI. The estimated glomerular filtration rate has become accepted as a more accurate indicator of renal function. We will externally validate the RCRI in a modern cohort of patients undergoing non-cardiac surgery and update its renal component.

**Methods and analysis:**

The Vascular Events in Non-cardiac Surgery Patients Cohort Evaluation (VISION) study is an international prospective cohort study. In this prespecified secondary analysis of VISION, we will test the risk estimation performance of the RCRI in ∼34 000 participants who underwent elective non-cardiac surgery between 2007 and 2013 from 29 hospitals in 15 countries. Using data from the first 20 000 eligible participants (the derivation set), we will derive an optimal threshold for dichotomising preoperative renal function quantified using the Chronic Kidney Disease Epidemiology Collaboration (CKD-Epi) glomerular filtration rate estimating equation in a manner that preserves the original structure of the RCRI. We will also develop a continuous risk estimating equation integrating age and CKD-Epi with existing RCRI risk factors. In the remaining (approximately) 14 000 participants, we will compare the risk estimation for cardiac complications of the original RCRI to this modified version. Cardiac complications will include 30-day non-fatal myocardial infarction, non-fatal cardiac arrest and death due to cardiac causes. We have examined an early sample to estimate the number of events and the distribution of predictors and missing data, but have not seen the validation data at the time of writing.

**Ethics and dissemination:**

The research ethics board at each site approved the VISION protocol prior to recruitment. We will publish our results and make our models available online at http://www.perioperativerisk.com.

**Trial registration number:**

ClinicalTrials.gov NCT00512109.

**Strengths and limitations of this study**

- The authors will externally validate the popular Revised Cardiac Risk Index (RCRI) for estimating risk of major adverse cardiac complications (including myocardial infarction, cardiac arrest and cardiac death) and update its renal component to use a threshold of estimated glomerular filtration rate instead of serum creatinine.
- The authors will also develop and validate a continuous risk estimating equation based on components of the RCRI in addition to age and estimated glomerular filtration rate.
- The analyses will be based on an estimated 34 000 patients (temporally independent samples of 20 000 for development and 14 000 for validation) from a large international prospective cohort study with systematic surveillance for major perioperative cardiac complications including myocardial infarction, cardiac arrest and cardiac death.
- Measurement of preoperative serum creatinine is based on routine practice, and some values will likely be measured using assays not calibrated to current reference standards.
- The loss to follow-up is expected to be <1% between discharge from hospital and 30 days after surgery and <7% of patients are expected to be missing preoperative data (which will be imputed to minimise risk of bias).

## Introduction

Globally, over 300 million people undergo non-cardiac surgery every year.^1^ Cardiac complications occur commonly after major non-cardiac surgery as a result of increased stress, inflammation, hypercoagulability and hypoxaemia induced by these procedures^2^ and are substantially more likely to occur in some patients than in others. On the basis of data collected from 1989 to 1994 at a single academic hospital, the Revised Cardiac Risk Index (RCRI)^3^ is widely used for perioperative cardiac risk stratification. It was developed for application in the setting of major elective non-cardiac surgery and estimates the risk of major postoperative cardiac complications until hospital discharge using six major components: the type of surgery (high vs low risk), history of ischaemic heart disease, history of congestive heart failure, history of stroke or transient ischaemic attack, history of diabetes requiring preoperative insulin and presence of preoperative renal impairment defined as serum creatinine >2 mg/dL (177 µmol/L).^3^ RCRI criteria are summarised in table 1.

**Table 1:** Predictor components of Revised Cardiac Risk Index (from Lee *et al*^3^) and corresponding VISION adaptation

Revised Cardiac Risk Index predictors	VISION adaptation
1. History of ischaemic heart disease	History of ischaemic heart disease
2. History of congestive heart failure	History of congestive heart failure
3. History of cerebrovascular disease (stroke or transient ischaemic attack)	History of cerebrovascular disease (stroke or transient ischaemic attack)
4. History of diabetes requiring preoperative insulin use	History of diabetes requiring preoperative insulin use
5. Preoperative creatinine >2 mg/dL	Preoperative creatinine >2 mg/dL
6. Undergoing high risk surgery (suprainguinal vascular, intraperitoneal or intrathoracic surgery)	Undergoing high risk surgery (thoracic aorta reconstruction, aortoiliac reconstruction, peripheral vascular reconstruction without aortic cross-clamping, extracranial cerebrovascular surgery, complex visceral resection, partial or total colectomy or stomach surgery, other intra-abdominal surgery, pneumonectomy, lobectomy, other thoracic surgery)

VISION, Vascular Events in Non-cardiac Surgery Patients Cohort Evaluation.

The RCRI allocates one point to the presence of each risk factor. While this has advantages in creating a simple to assess score, it also is likely to introduce inaccuracies. For example, the RCRI allocates one point if the serum creatinine threshold is crossed, regardless of age, sex and race; however, variations in these factors may result in very different estimated glomerular filtration rates (eGFR). For example, a serum creatinine of 2 mg/dL equates to an eGFR of 32 mL/min/1.73 m^2^ in a 75-year-old Caucasian male and 24 mL/min/1.73 m^2^ in a Caucasian female of the same age, when estimated using the Chronic Kidney Disease Epidemiology Collaboration (CKD-Epi) equation.^4^ Further, a recent meta-analysis suggests that the risk of early mortality after surgery begins to rise more steeply once eGFR falls below 60 mL/min/1.73 m^2^,^5^ which suggests that the threshold defining renal impairment in the RCRI may not be optimal.

The goals of our analyses will be (1) to validate the RCRI in a large international cohort of patients undergoing non-cardiac surgery, (2) to update its definition of preoperative renal impairment without undertaking any structural revision of this simple risk index and (3) to develop a risk equation that adds age and eGFR as continuous measures to existing RCRI risk factors for use in electronic risk calculators. This may improve the RCRI's performance in cardiac risk estimation and in risk stratification in the contemporary non-cardiac surgery setting.

## Methods

**Figure 1:**
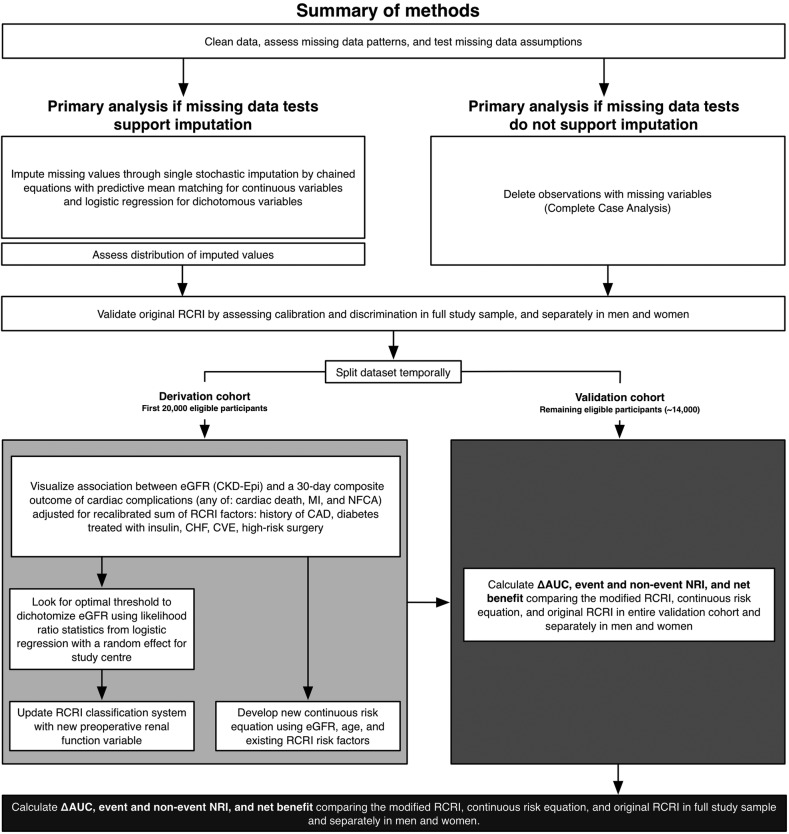
Summary of prespecified analysis plan. RCRI, Revised Cardiac Risk Index; CAD, coronary artery disease; CVE, cerebrovascular event; CHF, congestive heart failure; eGFR, estimated glomerular filtration rate; CKD, chronic kidney disease; MI, myocardial infarction; NFCA, non-fatal cardiac arrest; ΔAUC, change in Area Under the Receiver Operating Characteristics curve; NRI, categorical Net Reclassification Index.

Figure 1 summarises the plan for this prespecified secondary analysis of the Vascular Events In Non-cardiac Surgery Patients Cohort Evaluation (VISION) study. We have examined an early portion of the derivation data to assess the approximate number of events and the distribution of predictors and missing data; the validation data were not available for analysis when we wrote this protocol.

### Cohort definition

VISION is a prospective international cohort study that enrolled over 40 000 participants between August 2007 and October 2013 from 29 hospitals in 15 countries (ClinicalTrials.gov NCT00512109). VISION enrolled patients ≥45 years old and who underwent inpatient non-cardiac surgery that required general or regional anaesthetic.

Patients were screened for the study sequentially. They were identified by research personnel who screened daily patient lists in preoperative assessment clinics, on surgical wards, and in intensive care units; daily and previous-day surgical lists and patients in preoperative holding areas. In centres where the surgical volume exceeded the research staff's capacity to enrol consecutive patients, the centres were assigned random weeks for recruitment of all or randomly selected surgical services. Eligible consenting patients answered a series of questions about their past medical, surgical and social history. Moreover, study personnel reviewed patients' medical charts for further background history. Throughout each patient's hospital stay, research personnel performed clinical evaluations, reviewed medical records and noted outcome events. Outcomes were obtained from routine medical records and a follow-up telephone interview conducted with the patient or their caregiver 30 days after surgery. If the interview indicated the occurrence of an outcome, their physicians were contacted to obtain documentation.

Research staff at participating centres submitted the case report forms and supporting documentation directly to the data management system (iDataFax, coordinating centre, McMaster University, Hamilton, Ontario, Canada). Data monitoring involved central data consistency checks, statistical monitoring and onsite monitoring for all centres. For on-site monitoring, the central coordinator randomly selected participants with and without a perioperative complication; independent monitors audited their medical records and all other supporting documents.

We will present a flow diagram indicating the number of patients who fulfilled VISION eligibility criteria, were screened in time to participate, were enrolled and were included in the analyses, along with reasons for exclusion at each stage. The RCRI was developed and tested in patients undergoing elective procedures; thus, we will exclude from our analyses patients who underwent urgent or emergency surgery. Emergency surgery was defined in VISION as surgery that occurred <24 hours after a patient developed an acute surgical condition; urgent surgery occurred 24–72 hours after a patient developed an acute surgical condition. Patients and physicians most commonly consider additional testing and make decisions about whether to undergo a surgical procedure in non-urgent situations. Limiting our analyses to elective surgery additionally safeguards against misclassifying patients whose elevated preoperative creatinine represents evolving acute kidney injury instead of chronic renal impairment. Among the first 16 079 participants, 85.6% underwent an elective procedure.

Our primary outcome is a composite of 30-day major cardiac complications, including 30-day non-fatal myocardial infarction (according to its universal definition^6^), non-fatal cardiac arrest and death due to cardiac causes.^2^ The online supplementary web appendix provides definitions for these outcomes.

10.1136/bmjopen-2016-013510.supp1 supplementary appendix

### Descriptive statistics

We will present a table (analogous to table 2) describing our sample's distribution of outcomes and RCRI risk factors, eGFR, age, country and type of surgery (major general surgery, major neurosurgery, major thoracic surgery, major vascular surgery, major orthopaedic surgery, major urogenital surgery and low risk surgery). Definitions for these variables are provided in the online supplementary web appendix.

**Table 2:** Summary of participant characteristics in full study sample

Characteristics	Total	Any cardiac complication	RCRI class			
	No. (% of total)	No. (%)	Class I No. of events (% and 95% CI)/No. in class (%)	Class II No. of events (% and 95% CI)/No. in class (%)	Class III No. of events (% and 95% CI)/No. in class (%)	Class IV No. of events (% and 95% CI)/No. in class (%)
No. of total participants (%)						
Any cardiac complications	–	–				
Cardiac death						
Myocardial infarction						
Non-fatal cardiac arrest						
Age, years						
45–64						
65–74						
75+						
Women						
RCRI risk factors						
History of CAD						
History of CVE						
History of CHF						
Diabetes treated with insulin						
High risk surgery						
Serum creatinine >2mg/dL						
Preoperative CKD-Epi eGFR, mL/min/1.73 m^2^						
120+						
90–119						
60–89						
45–59						
30–44						
15–29						
<15 or dialysis						
Type of surgery						
Major general						
Major neurological						
Major thoracic						
Major vascular						
Major orthopaedic						
Major urogenital						
Low risk only						
Country						
Canada						
USA						
Columbia						
Peru						
Brazil						
UK						
Poland						
Spain						
France						
India						
Malaysia						
Hong Kong						
Australia						
South Africa						
Italy						

We will similarly summarise the data separately for the derivation and validation samples and for the original and our modified RCRI. Original RCRI is based on Lee *et al*.^3^

CAD, coronary artery disease; CHF, congestive heart failure; CKD-Epi, Chronic Kidney Disease Epidemiology Collaboration equation; CVE, cerebrovascular events (stroke or transient ischaemic attack); eGFR, estimated glomerular filtration rate; RCRI, Revised Cardiac Risk Index.

### Approach to missing data

VISION made extensive efforts to enrol and prospectively follow a representative sample of patients undergoing non-cardiac surgery. Few patients had missing data on preliminary examination of the first 13 766 potentially eligible participants. Please refer to the online supplementary web appendix for a more complete discussion of our approach to missing data, which we briefly summarise here.

Preoperative creatinine values were missing in 6.5% of participants who were not already receiving dialysis before surgery. Using multivariable logistic regression, we will examine adjusted associations between creatinine measurement and observed covariates to test fundamental assumptions about the mechanism by which data are missing. We will use single stochastic conditional imputation with predictive mean matching^7^ for creatinine and logistic regression for any other missing RCRI variables, both performed with fully conditional specification.^8^

All variables involved in the RCRI will be included in the imputation model to avoid introducing bias in the main analysis. We will also include the following auxiliary variables that are not part of the RCRI but are potentially related to the value of the missing creatinine or to the propensity for measurement: age in years (modelled linearly), sex, requiring assistance with activities of daily living, history of peripheral vascular disease, history of atrial fibrillation, history of hypertension, active cancer, history of chronic obstructive pulmonary disease, whether the procedure was a vascular surgery, general surgery, thoracic surgery, orthopaedic surgery, urogenital surgery or neurosurgery, the study centre as an important determinant of preoperative assessment practice, ethnicity and a term for a potential age×sex interaction. Including variables that are not truly related to the missing variable does not affect the validity of the imputation procedure while including many potential factors in the imputation model makes imputation assumptions more plausible.^9^ We will also include the composite outcome (30-day cardiac complications) in the imputation model; failing to include the outcome in imputation of covariates can substantially dilute its relationship with the imputed variables.^10^ We will present a kernel density diagnostic plot comparing the distribution of the imputed and available values of serum creatinine.

A small proportion (∼0.3%) of patients were lost-to-follow-up before 30 days after their surgery and did not experience a cardiac complication before hospital discharge; they will be included in the analysis but are censored at the time of discharge. A very small proportion of the first 13 766 (∼0.2%) were missing data on ethnicity, which is required to calculate eGFR. For these patients, we will assign the ethnicity most common to patients recruited at their respective study centre.

### External validation of the RCRI

We will test the calibration and discrimination of the original RCRI in the full sample of eligible participants. A risk prediction model is said to be well calibrated when the event probabilities that it predicts for different groups of patients closely approximate the observed proportion of patients who truly experienced events in those groups. The RCRI is a classification system based on summation of risk factors.^3^ Using a revised composite outcome definition consistent with ours (including myocardial infarction, cardiac arrest and cardiac death) in the original RCRI cohort, Devereaux *et al*^2^ showed that 59 of 4315 participants experienced an event; estimated risks and their 95% CIs across RCRI classes I, II, III and IV were 0.4% (0.1% to 0.8%), 1.0% (0.5% to 1.4%), 2.4% (1.3% to 3.5%) and 5.4% (2.8% to 7.9%), respectively. We will calculate class-specific event rates along with 95% CIs and will assess discrimination of the original RCRI by calculating the area under the receiver operating characteristics curve (AUC).

### Updating the definition of preoperative renal impairment

No new risk factors will be introduced in this analysis, weights for existing RCRI risk factors will remain the same (1 point awarded per factor), and the four-class system will remain intact. We will use the CKD-Epi equation^4^ to calculate eGFR. This equation has become widely accepted in the staging of chronic kidney disease^11^ and is increasingly reported automatically along with serum creatinine by many laboratories.

#### Definitions

- RCRI_Original_: Sum of the original RCRI risk factors, calculated for each patient. This will range from 0 to 6.
- β_cal_: Calibration slope for RCRI_Original_.
- RCRI_OriginalRecal_: Recalibrated sum of RCRI risk factors, calculated for each patient. This will range from 0 to 6 and will have a slope of 1 in a logistic regression against the composite outcome of cardiac complications.
- RCRI_NoRenal_: Sum of the original RCRI risk factors excluding renal impairment, calculated for each patient after recalibration. This will range from 0 to 5.
- NewRenal: A new variable expressing renal function in eGFR calculated with the CKD-Epi equation.
- RCRI_RenalRevised_: Sum of RCRI risk factors, including the updated renal impairment variable.

#### Recalibration

We will estimate a calibration slope, β_cal_, for the original RCRI risk factor sum in the following equation using logistic regression with a random intercept for study centre:
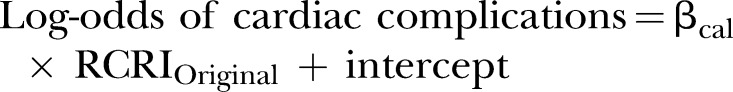


We will then calculate the recalibrated RCRI score for each patient by multiplying RCRI_Original_ by β_cal_. We term this recalibrated index RCRI_OriginalRecal_.

#### Exploratory data visualisation

We will model the relationship between eGFR (in its continuous form, expressed with restricted cubic splines) and log-odds of major cardiac complications with a mixed-effects logistic regression model, adjusted for RCRI_NoRenal_ (with slope constrained to equal 1) and a random intercept for study centre.

#### Procedure to identify the statistically optimal dichotomisation threshold for eGFR in the RCRI

1. Create 10 versions of the variable NewRenal, each dichotomising eGFR at a different threshold ranging from 15 to 60 mL/min/1.73 m^2^ in increments of 5 mL/min/1.73 m^2^, with those patients who were already receiving dialysis preoperatively placed in the lowest category.
2. Sequentially test the relationship between each NewRenal variable and 30-day cardiac complications using multivariable logistic regression adjusted for RCRI_NoRenal_. In these analyses, the slope of RCRI_NoRenal_ will be constrained to 1 to prevent re-estimation of the slopes of the other RCRI variables. This will allow us to redefine the renal component without altering the weights of the other components.
3. Identify the threshold(s) for which NewRenal has slope β_1_ not significantly different from 1 according to a two-tailed likelihood ratio χ^2^ test with p>0.05. If multiple thresholds meet this criterion, we will select the one that results in the highest model likelihood ratio statistic compared to a model with no renal information. The selected variable is NewRenal_optimal_.
4. If the threshold selected for NewRenal_optimal_ is not one of 15, 30, 45 or 60 mL/min/1.73 m^2^, compare NewRenal_optimal_ to the nearest of these four thresholds using the likelihood ratio χ^2^ test. If p>0.05, make that closest threshold NewRenal_optimal_.
5. Replace the renal impairment term in RCRI_OriginalRecal_ with NewRenal_optimal_ to calculate RCRI_RenalUpdate_.
6. Classify patients in one of four RCRI classes using RCRI_RenalUpdate_, so that those with 0 risk factors are assigned to RCRI Class I; 1 risk factor, Class II; 2 risk factors, Class III; and 3 or more risk factors, Class IV.

### Development of a new risk equation

With logistic regression, we will develop a continuous risk estimating equation that adds age, eGFR and the interaction between them, to existing RCRI risk factors. We will model continuous variables using restricted cubic spline functions to allow for non-linearity and will present the resultant model as an equation that can be integrated into risk calculation software for use on the web, on mobile devices, and in clinical information systems.

### Evaluation of predictive performance

In the validation cohort, we will compare the prediction performance of the original RCRI classification system to the updated systems using established methods,^12^ including reclassification tables,^13^ change in area under the receiver operating characteristics curve (ΔAUC, where a ΔAUC represents significantly better discrimination performance if the lower bound of its 95% CI exceeds 0) and the categorical Net Reclassification Index (NRI) with three risk categories (<5%, 5–15% and >15%). These categories have been used in previous publications,^14–16^ and we believe that patients and practitioners find them relevant. In sensitivity analyses, we will use four risk categories (<5%, 5–9.9%, 10–15% and >15%), as well as the risk categories recommended in the European Society of Cardiology guidelines (<1%, 1–5% and >5%).^17^

We will calculate categorical NRI separately for people who did and did not experience cardiac complications. For those who did, we will assign 1 for reclassification to a higher risk category, −1 for reclassification to a lower category and 0 for no change in category. We will do the opposite for people who did not experience cardiac complications, awarding 1 for downward classification and −1 for upward classification. We will then divide the sum of the individual scores by the number of people in each group.^18^ ^19^ We will also assess each model's potential for clinical usefulness using net benefit and decision curve analysis.^20^ ^21^ This decision-analytic method estimates the clinical net benefit of prediction models as the sum of the harms (false positives) subtracted from the sum of the benefits (true positives), where harm is weighted by a factor relating the risk threshold at which a patient or practitioner might choose to modify treatment or investigation. A valuable model has high net benefit across the relevant range of these risk thresholds. We will then perform these performance comparisons separately for men and women and repeat them in the full study sample.

### Sample size

Validation and updating of prediction models require fewer events than development of a new model.^9^ ^22^ ^23^ However, the required sample size for a validation study is unclear. Guidance on this matter suggests that at least 100 events and 100 non-events are required, but most prospective validation studies are far smaller.^24^ Guidance regarding sample size for model development and updating suggests that at least 10 events per parameter estimated (including regression slopes and intercept) may be necessary to avoid serious problems with statistical overfitting and that some residual overfitting may remain with fewer than 20 events per variable.^23^ ^25^ We estimate that ∼500 events will occur in our derivation sample of ∼20 000 participants. This will allow for over 40 events per parameter estimated in the simple modification of the RCRI classification system and at least 20 events per parameter for the development of the continuous risk equation. We will assess the performance of our models in a temporally independent sample of 14 000 participants with an estimated 350 events not used in derivation; thus, we can be confident in the generalisability of the thresholds we discover and the performance characteristics we estimate.

## Presentation of results

We will present the results of our analyses in a table analogous to table 3.

**Table 3:** Proposed summary of performance comparisons

Performance metrics Validation sample	Original RCRI			eGFR-modified RCRI			Continuous risk equation		
	Overall	Women	Men	Overall (Δoriginal)	Women (Δoriginal)	Men (Δoriginal)	Overall (Δoriginal/Δmodified)	Women (Δoriginal/Δmodified)	Men (Δoriginal/Δmodified)
AUC (95% CI)									
NB_5%_ (95% CI)									
NB_10%_ (95% CI)									
NB_15%_ (95% CI)									
NRI_events_ (95% CI)	–	–	–						
NRI_nonevents_ (95% CI)	–	–	–						
Full sample									
AUC (95% CI)									
NB_5%_ (95% CI)									
NB_10%_ (95% CI)									
NB_15%_ (95% CI)									
NRI_events_ (95% CI)	–	–	–						
NRI_nonevents_ (95% CI)	–	–	–						

Original RCRI is based on Lee *et al*.^3^

AUC, Area Under the Receiver Operating Characteristics curve; NB, Net Benefit; NRI, 3-category Net Reclassification Index (<5%, 5–15% and >15%); RCRI, Revised Cardiac Risk Index.

## Discussion

Given the global volume of non-cardiac surgery and large contribution of cardiac complications to overall perioperative mortality, effective strategies that inform risk-based decisions in perioperative care are essential. Owing to its simplicity, the RCRI has become a very popular risk stratification system for patients undergoing elective non-cardiac surgery. However, a systematic review of 24 studies validating the RCRI found that only 5 relevant high-quality validation studies enrolling a total of 2046 participants have been published.^26^ We have prespecified a plan to externally validate the RCRI and to update its definition of preoperative renal impairment. No new risk factors will be introduced in the simple update, weights for existing RCRI risk factors will remain the same (1 point per factor), and the four-class system will remain intact. If successful, we will improve the RCRI's cardiac risk stratification performance in the contemporary non-cardiac surgery setting without undertaking any structural revision of this popular risk index. Our hope is that this approach will facilitate the use of the modified RCRI in clinical practice. To gain further predictive accuracy, we will integrate the CKD-Epi eGFR estimating equation and current RCRI risk factors into a new equation that can be implemented in risk calculation software for use on the web, on mobile devices and in clinical information systems.

We anticipate to see substantially higher risk of events across RCRI classes than was observed in the original publication by Lee *et al.*^3^ First, the RCRI was originally developed and validated with data from a single centre collected between 1989 and 1994; patients were followed until discharge from hospital.^3^ While most events occur in the immediate postoperative period, we included outcomes occurring up to 30 days postoperatively because this timeframe is relevant for decision-making and the emphasis and incentives for early discharge in some settings may otherwise have caused us to miss a substantial number of events. Second, there is evidence that patients undergoing surgery today have a higher baseline risk of events because they are, on average, older and suffer from more comorbid conditions.^27^ Third, current troponin-based tests are more sensitive for diagnosing myocardial infarction than the creatine kinase-based tests used in the original study and are likely to identify a greater number of prognostically relevant postoperative events.

There exist some other minor differences between RCRI outcomes and our study. The RCRI's original composite outcome definition included pulmonary oedema and complete heart block; these outcomes were not collected in VISION explicitly. Pulmonary oedema is instead captured as a potential sign of myocardial infarction, which also requires a characteristic rise or fall in troponin (see online supplementary web appendix). Pulmonary oedema in the absence of troponin changes likely reflects a state of fluid overload and not true new-onset cardiomyopathy. Although we did not collect data regarding complete heart block, it occurred in only 4 of the 4315 patients in the original RCRI publication (2 in the derivation cohort and 2 in the development cohort) and is unlikely to affect the validity of our analysis. Further, the original RCRI outcome definition did not include death due to cardiac causes. This outcome occurred in 0.3% of patients; we will include this important outcome in our composite definition. The outcomes we selected are consistent with prior analyses of major postoperative cardiac complications.^2^

The accuracy of creatinine-based GFR estimating equations dependents on the calibration of the serum creatinine assay to reference standards used in the development of the GFR estimation equations. Uncalibrated assays suffer from a constant bias that overestimates creatinine and underestimates eGFR compared to their calibrated counterparts.^28–30^ It is likely that some laboratories in the VISION study do not use standardised creatinine assays. Creatinine values are not always measured by the participating centre but may have been measured by private laboratories before the patient was admitted for surgery. Calibration bias may lead us to identify as ‘optimal’ an eGFR dichotomisation threshold that is lower than would be optimal when creatinine is measured with a calibrated assay. The impact of this calibration bias on eGFR is minimal at high serum creatinine (severely impaired kidney function) but becomes particularly pronounced at the high normal range corresponding to moderately reduced GFR (≥60 mL/min/1.73 m^2^).^29^ Thus, we will search for optimal cutpoints in the range of eGFR ≤60 mL/min/1.73 m^2^.

Major cardiac complications are common after non-cardiac surgery. To make decisions about the appropriateness of surgery and next steps in management, clinical practice guidelines recommend assessment of preoperative risk beginning with clinical risk indices.^17^ ^31^ Using prospectively collected data from a large international study, these analyses will validate the popular RCRI and update its use of preoperative renal impairment for the contemporary surgical setting.
