# Supplementary material for: External validation of the Revised Cardiac Risk Index and update of its renal variable to predict 30-day risk of major cardiac complications after non-cardiac surgery: rationale and plan for analyses of the VISION study
Source: BMJ Open. 2017 Jan 9;7(1):e013510. doi: 10.1136/bmjopen-2016-013510 (PMC5223708; doi:10.1136/bmjopen-2016-013510)
Supplement: supplementary appendix [file bmjopen-2016-013510supp_appendix.pdf]

Web Appendix

Definition of major cardiac complications as a composite of myocardial infarction, nonfatal cardiac arrest, and cardiac death 2

Type of surgery performed 3

Clinical factors 5

List of study centres 6

Justification of approach to missing data 7

The imputation method 8

References 11

Definition of major cardiac complications as a composite of myocardial infarction, nonfatal cardiac arrest, and cardiac death

1. Myocardial infarction defined as any one of the following criteria (A, B or C) according to its universal definition[6]:

A. A typical rise of troponin or a typical fall of an elevated troponin detected at its peak post surgery in a patient without a documented alternative explanation for an elevated troponin (e.g., pulmonary embolism). This criterion also required **that 1 of the following** must also exist:

i. ischemic signs or symptoms (i.e., chest, arm, neck or jaw discomfort; shortness of breath; pulmonary edema), **OR**

ii. development of pathologic Q waves present in any two contiguous leads that are ≥ 30 milliseconds, **OR**

iii. ECG changes indicative of ischemia (i.e., ST segment elevation [≥ 2 mm in leads V1, V2, or V3 OR ≥ 1 mm in the other leads], ST segment depression [≥ 1 mm], or symmetric inversion of T waves ≥ 1 mm) in at least two contiguous leads, **OR**

iv. coronary artery intervention (i.e., PCI or CABG surgery), **OR**

v. new or presumed new cardiac wall motion abnormality on echocardiography or new or presumed new fixed defect on radionuclide imaging.

B. Pathologic findings of an acute or healing myocardial infarction

C. Development of new pathological Q waves on an ECG if troponin levels were not obtained or were obtained at times that could have missed the clinical event.

2. Nonfatal cardiac arrest – Nonfatal cardiac arrest was defined as successful resuscitation from either documented or presumed ventricular fibrillation, sustained ventricular tachycardia, asystole, or pulseless electrical activity requiring cardiopulmonary resuscitation, pharmacological therapy, or cardiac defibrillation.

3. Cardiac death – Death thought to be due to a cardiac cause including myocardial infarction, asystole, ventricular fibrillation, pulseless electrical activity, other sudden or arrhythmic death, sustained ventricular tachycardia, cardiogenic shock, congestive heart failure, or other cause thought to be cardiac in nature.

Type of surgery performed

If a patient underwent more than one surgery, all performed surgeries were included. If patients underwent any of the major surgical procedures, they were not classified as undergoing a 'low risk surgery'.

Major Vascular Surgery

1. Thoracic aorta reconstructive vascular surgeries (thoracic aortic aneurysm repair, repair of supra-aortic trunks not requiring total cardiopulmonary bypass, thoracoabdominal aortic aneurism repair with or without aorto-femoral bypass)

2. Aorto-iliac reconstructive vascular surgery (open abdominal aortic aneurysm repair, aorto-femoral bypass, iliac-femoral bypass, renal artery revascularization, celiac artery revascularization, superior mesenteric artery revascularization)

3. Peripheral vascular reconstruction without aortic cross-clamping (axillo-femoral bypass, femoral-femoral bypass, femoro-infragenicular bypass, profundoplasty, or other angioplasties of the infrainguinal arteries)

4. Extracranial cerebrovascular surgery (carotid endarterectomy, carotid-subclavian bypass)

5. EVAR – endovascular abdominal aortic aneurysm repair

Major General Surgery

1. Complex visceral resection (surgery involving the liver, esophagus, pancreas, or multiple organs)
2. Partial or total colectomy or stomach surgery
3. Other intra-abdominal surgery (gallbladder, appendix, adrenals, spleen, regional lymph node dissection)
4. Major head and neck resection for non-thyroid tumor

Thoracic Surgery
1. Pneumonectomy
2. Lobectomy
3. Other thoracic (wedge resection of lung, resection of mediastinal tumor, major chest wall resection)

Major Urology or Gynecology Surgery
1. Visceral resection (nephrectomy, ureterectomy, bladder resection, retroperitoneal tumor resection, exenteration [i.e. radical procedure for cancer to remove pelvic organs])
2. Cytoreductive surgery "debulking" done when cancer has spread in the pelvic/abdominal area, to remove as much of the tumor as possible
3. Radical hysterectomy is surgery to remove the uterus, cervix and part of the vagina
4. Hysterectomy is surgery to remove the uterus and usually the cervix
5. Radical prostatectomy is surgery to remove entire prostate gland and surrounding tissue
6. Transurethral prostatectomy to remove overgrowth of prostate tissue

Major Orthopedic Surgery
1. Major hip or pelvic surgery (hemi or total hip arthroplasty, internal fixation of hip, pelvic arthroplasty)
2. Internal fixation of femur

3. Knee arthroplasty
4. Above knee amputations
5. Lower leg amputation (amputation below knee but above foot)

Major Neurosurgery
1. Craniotomy
2. Major spine surgery is surgery involving multiple levels of the spine.

Low Risk Surgeries (parathyroid, thyroid, breast, hernia, local anorectal procedure, oopherectomy, salpingectomy, endometrial ablation, peripheral nerve surgery, ophthalmology, ears/nose/throat surgery, vertebral disc surgery, hand surgery, cosmetic surgery, arterio-venous access surgery for dialysis, other surgeries).

Urgent or emergency surgeries: surgeries performed within 72 hours of acute event that led to need for surgery

Clinical factors

Age (for descriptive purposes only, not part of RCRI) – the patient's age in years, calculated as the difference between their birthdate and the date of surgery and rounded down to the nearest year.

History of congestive heart failure – A physician diagnosis of a current or prior episode of congestive heart failure or prior radiographic evidence of vascular redistribution, interstitial pulmonary edema, or frank alveolar pulmonary edema.

History of coronary artery disease – A current or prior history of **any one** of the following:

i. angina
ii. myocardial infarction or acute coronary syndrome
iii. a segmental cardiac wall motion abnormality on echocardiography or a segmental fixed defect on radionuclide imaging
iv. a positive radionuclide exercise, echocardiographic exercise, or pharmacological cardiovascular stress test demonstrating cardiac ischemia
v. coronary angiographic or CT coronary angiographic evidence of atherosclerotic stenosis ≥50% of the diameter of any coronary artery
vi. ECG with pathological Q waves in two contiguous leads

History of cerebral vascular event – A physician diagnosis of stroke, CT or MRI evidence of a prior stroke, or physician diagnosis of a prior transient ischemic attack.

Diabetes requiring preoperative insulin – Patient states they have been diagnosed with diabetes or a physician has previously recorded that the patient has diabetes. This includes current gestational diabetes, but not past gestational diabetes that has resolved. The patient was also taking insulin prior to surgery.

Clinical factors

Age (not part of RCRI) – the patient's age in years, calculated as the difference between their birthdate and the date of surgery and rounded down to the nearest year.

History of congestive heart failure – A physician diagnosis of a current or prior episode of congestive heart failure or prior radiographic evidence of vascular redistribution, interstitial pulmonary edema, or frank alveolar pulmonary edema.

History of coronary artery disease – A current or prior history of **any one** of the following:

i. angina
ii. myocardial infarction or acute coronary syndrome
iii. a segmental cardiac wall motion abnormality on echocardiography or a segmental fixed defect on radionuclide imaging
iv. a positive radionuclide exercise, echocardiographic exercise, or pharmacological cardiovascular stress test demonstrating cardiac ischemia
v. coronary angiographic or CT coronary angiographic evidence of atherosclerotic stenosis ≥50% of the diameter of any coronary artery
vi. ECG with pathological Q waves in two contiguous leads

History of cerebral vascular event – A physician diagnosis of stroke, CT or MRI evidence of a prior stroke, or physician diagnosis of a prior transient ischemic attack.

Diabetes requiring preoperative insulin – Patient states they have been diagnosed with diabetes or a physician has previously recorded that the patient has diabetes. This includes current gestational diabetes, but not past gestational diabetes that has resolved. The patient was also taking insulin prior to surgery.

List of study centres

McMaster University Medical Centre	Hamilton, Canada
Juravinski Hospital	Hamilton, Canada
Hamilton General Hospital	Hamilton, Canada
Saint Joseph's Hospital	Hamilton, Canada
Walter C. Mackenzie Health Sciences Centre	Edmonton, Canada
Winnipeg Health Sciences Centre	Winnipeg, Canada
Prince of Wales Hospital	Hong Kong, China
Victoria Hospital	London ON, Canada
Hospital Universitario de Santander	Bucaramanga, Colombia
Foundation CardioInfanil	Bogota, Colombia
HCOR (Hospital do Coracao)	Sao Paulo, Brazil
Hospital de Clinicas de Porto Alegre	Porto Alegre, Brazil
Hospital Nacional Cayetano Heredia	Lima, Peru
Hospital de Sant Pau	Barcelona, Spain
Hospital Gregorio Marañon	Madrid, Spain
Barts And The London	London, UK
University College Hospital	London, UK
Leeds Teaching Hospitals	Leeds, UK
Royal Liverpool University Hospital	Liverpool, UK
Pitie-Salpetriere Hospital	Paris, France
St. John's Medical College	Bangalore, India
Christian Medical College	Ludhiana, India
University Malaya Medical Centre	Kuala Lumpur, Malaysia
Italian National Cancer Institute Regina Elena	Rome, Italy
Inkosi Albert Luthuli Hospital	Durban, South Africa
Westmead Hospital	Sydney, Australia
Jagiellonian University Medical College	Krakow, Poland
Washington University School of Medicine	St. Louis, USA
Cleveland Clinic	Cleveland, USA

Justification of approach to missing data

Serum creatinine information is collected as part of routine care and may be missing under one of three general mechanisms:

1. Missing completely at random (MCAR): The creatinine value was lost or practitioners forgot to order it for reasons independent of the would-be creatinine value or any other observed or unobserved covariate.

2. Missing at random (MAR): The creatinine value was not measured for reasons unrelated to the would-be creatinine value itself but propensity for measurement is related to other observed covariates. For example, a low risk procedure, young age, or absence of cardiovascular or metabolic comorbidities may have led practitioners to assume normal kidney function and abstain from further assessment. Local policies for preoperative assessment may also influence the practice of measuring preoperative creatinine.

3. Missing not at random (MNAR): The measurement of preoperative creatinine is dependent on the would-be creatinine value itself. Mild and moderate renal impairment is commonly without signs and symptoms. It is possible that people without measured creatinine values actually have creatinines reflective of normal kidney function or mild to moderate renal impairment. They may be less likely to have severe renal impairment because those patients are more likely to exhibit signs and symptoms that would prompt creatinine measurement.

By assessing the adjusted associations between creatinine measurement and observed covariates, we will test whether assuming the first mechanism (MCAR) is invalid (i.e., MCAR is not valid if such associations exist). However, there is not usually a way to determine if a variable is MAR or MNAR. In our study, two factors allow us to perform some limited testing for an MNAR mechanism: patients with

severe renal impairment almost always suffer from anemia, and the most severe cases of renal impairment are treated with dialysis. We will test whether the propensity for creatinine measurement was associated with either of these factors after adjusting for the remaining covariates and the study centre. If we find no association between preoperative hemoglobin (categorized as <9, 9-10, 10-12, >12 g/dL) or dialysis status and propensity for creatinine measurement after adjusting for the remaining covariates and the study centre, this would lend some support to the theory that creatinine measurement is not independently related to actual renal function and is therefore not MNAR.

The imputation method

Simulations studies demonstrate that a complete case analysis (i.e. an analysis that deletes observations with missing values) produces valid parameter estimates when data are MCAR or if the propensity for missingness is independent of the outcome after adjusting for the other covariates in the model of interest[1,2]. Under these condition, complete case analyses are unbiased and analyses based on (stochastic) conditional imputation, whether multiple or single, exhibit bias. The differences are expected to be less pronounced for small amounts of missing data and in large datasets.

If data are not MCAR and if missingness is not independent of the outcome after adjustment, parameter estimates can be badly biased. Even if the strict MCAR assumption holds, the analysis is inefficient because it decreases statistical power by discarding available data. These problems are less pronounced with small amounts of missing data (<10%) but can be substantial as the proportion of missing data grows.

Simple imputation methods such as using the mean value of the observed cases to replace the missing cases fail to relax the assumption that data is missing completely at random because they do not take into account relationships among the imputed value and covariates. Conditional mean imputation addresses this problem by estimating a mean value conditional on the remaining covariates with a

regression model. However, this approach (like simple mean imputation) is deterministic and fails to introduce variance in the imputed values as one would observe naturally. Stochastic conditional imputation addresses this problem by introducing random noise to the imputed conditional means by taking a random draw from the predicted distribution of imputed values. The random element reflects the natural uncertainty of the imputed values, which is especially important if this uncertainty is substantial.

Standard analyses with data imputed through all of these approaches treat the imputed values as though they were observed and ignore the uncertainty inherent in imputing unobserved values. This understates the variance of parameter estimates and produces confidence intervals with more narrow coverage than if this uncertainty had been accounted for, inflating the risk of Type 1 error. The degree to which this is a problem is related to the amount of imputed data: it is less of a concern with relatively small amounts of missing data and more with larger amounts. Large datasets (with >100 events) with a small proportion of missing values are the least affected[2]. Complex variance estimation methods exist to correct this situation but they have not been implemented in standard statistical software.

Multiple imputation addresses this limitation through a computation-intensive technique that uses conditional stochastic imputation to produce several (M) imputed datasets and, at the analysis stage, combines the results from analyses conducted in each dataset (e.g. the regression of interest) with formulas (Rubin's Rules) to produce confidence intervals that better capture the uncertainty inherent in imputation[3]. Implementations of multiple imputation in standard statistical software (e.g. SAS, Stata, and R) allow estimates from most standard analyses to be combined with Rubin's Rules. However, no such facilities are available for the more customized aspects involved in our analyses. In addition, the extensive use of resampling and data-splitting techniques integral to our analyses are already computationally intensive and become practically intractable with the use of multiple imputation.

Single stochastic conditional imputation is typically sufficient to provide unbiased point estimates of predictor effects and predictions, and to evaluate the performance of prediction model[2]. These are the primary goals in our analysis. Multiple imputation has somewhat of an advantage for estimating the uncertainty of the predictions (through accurate confidence intervals), but single imputation provides a reasonable approximation if the amount of missing data is small and the dataset is large[2].

We will use single stochastic conditional imputation with predictive mean matching for continuous variables and logistic regression for binary variables. Predictive mean matching is a semiparametric method that relies on the initial predictions from the stochastic conditional imputation model to define a similarity metric and uses a k-nearest neighbor algorithm (k=1 in our case) to find a patient with a patient with an observed value who is similar (according to the other covariates) to a patient with a missing data point, substituting the observed for the unobserved[4]. It is particularly useful if the data being imputed may not follow a normal distribution[4]. We will use the method of fully conditional specification to impute missing values iteratively[5].

References

1 White IR, Carlin JB. Bias and efficiency of multiple imputation compared with complete-case analysis for missing covariate values. *Stat Med* 2010;**29**:2920–31. doi:10.1002/sim.3944

2 Steyerberg EW. *Clinical Prediction Models: A Practical Approach to Development, Validation, and Updating*. New York, NY: Springer-Verlag New York 2009.

3 Little RJA, Rubin DB. *Statistical Analysis with Missing Data*. New York: : Wiley & Sons 1987.

4 Landerman LR, Land KC, Pieper CF. An Empirical Evaluation of the Predictive Mean Matching Method for Imputing Missing Values. *Sociol Methods Res* 1997;**26**:3–33. doi:10.1177/0049124197026001001

5 Van Buuren S, Oudshoorn C. MICE: multivariate imputation by chained equations. *J Stat Softw* 2010;**VV**.
